# The latitudinal patterns of phylogenetic, functional and numerical interaction specificity in flea–mammal networks vary between biogeographic realms

**DOI:** 10.1017/S0031182026102182

**Published:** 2026-06

**Authors:** Boris R. Krasnov

**Affiliations:** Mitrani Department of Desert Ecology, Swiss Institute for Dryland Environmental and Energy Research, Jacob Blaustein Institutes for Desert Research, Ben-Gurion University of the Negev, Sede Boqer Campus, Midreshet Ben-Gurion, Israel

**Keywords:** biogeographic realms, fleas, interaction specificity, latitude, small mammals

## Abstract

The latitudinal patterns of the phylogenetic, functional and numerical interaction specificity (PIS, FIS and NIS, respectively) of fleas and mammals in their interaction networks, from 4 biogeographic realms, were investigated at the intraspecific, interspecific and community levels to understand whether the spatial variation of interaction specificity followed the predictions of a latitudinal specialization gradient. In fleas, no latitudinal pattern of PIS was detected. Flea FIS varied with latitude in all realms at the intraspecific level and in 2 realms at the interspecific and community levels. Latitudinal trends in NIS were detected in all realms (except Neotropics) at the intraspecific level and in the Old World realms at both higher levels. In hosts, PIS varied with latitude in the Afrotropics and the Nearctic at the intraspecific level and in the Afrotropics at the interspecific and community levels. FIS demonstrated a latitudinal trend in the Nearctic at the intraspecific level and in the Palearctic at the community level. Relationships between latitude and NIS were found in all realms (except Neotropics) at the intraspecific level and in the Old World realms at the interspecific and community levels. The directions of the latitudinal pattern of the same specificity facet could be positive or negative. They differed between fleas and hosts, realms and organizational levels. The occurrence and direction of the latitudinal specialization gradient are explained by the combined actions of evolutionary, historical and ecological factors. Pooling data from regions with different biogeographical histories and landscape structures, for a macroecological analysis, may produce spurious results.

## Graphical abstract

**Figure:**
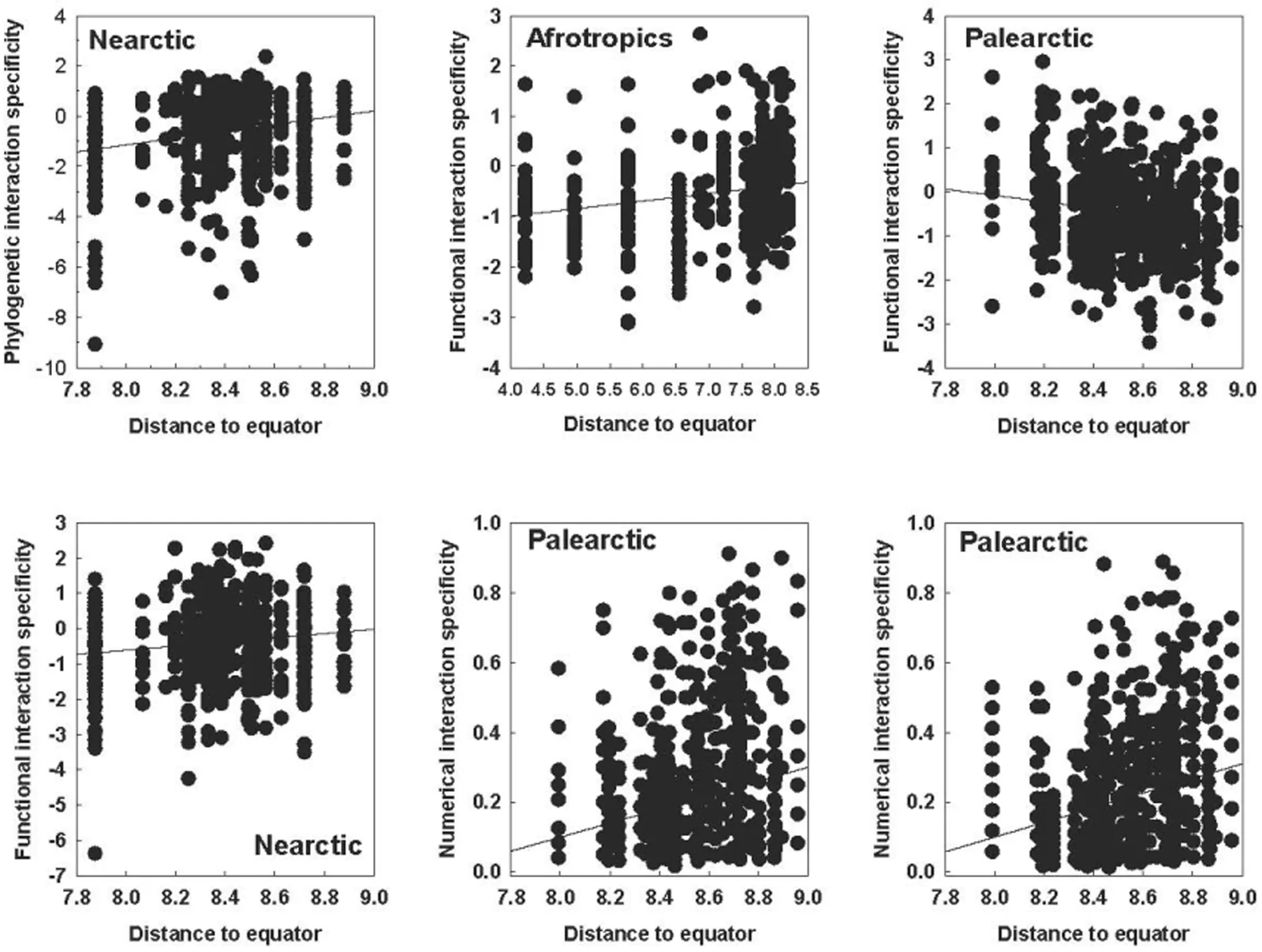

## Introduction

The latitudinal gradient of species diversity is one of the most famous and commonly recognized biogeographic patterns, with richer biological diversity occurring in tropical communities than in communities at higher (to the north of the equator) or lower (to the south of the equator) latitudes (Pianka, 1966; Rohde, 1992; Rosenzweig, 1995; Gaston and Blackburn, 2000). One of the consequences of higher species richness at lower latitudes has been thought to be the increased niche segregation of species, which, in turn, results in higher specialization (MacArthur, 1984). In other words, tropical species are expected to have narrower niches than species from temperate and polar regions (i.e. latitudinal specialization gradient). The mechanism of narrower niches and the resultant higher specialization in communities closer to the equator has been proposed to be an indirect effect of latitude on niche breadth through its effect on species richness (Vázquez and Stevens, 2024). An additional, not necessarily alternative, mechanism for broader niches in temperate and polar habitats could be the higher variability (e.g. due to seasonality) and the generally lower productivity of these habitats (Dalsgaard et al., 2011).

Starting from the pioneering analyses of Ollerton and Cranmer (2002) and Vázquez and Stevens (2004), the metrics of specialization developed in network ecology have been repeatedly used to investigate the variation in the degree of specialization along latitudes. The results of these studies were contradictory and demonstrated patterns either confirming the latitudinal specialization gradient (e.g., Dalsgaard et al., 2011) or opposing it (e.g. Dalsgaard et al., 2017) or finding no latitudinal pattern of specialization at all (Ten Caten and Dallas, 2024). Moreover, it appears that the relationship between the degree of specialization and latitude varies between taxa (Svensson-Coelho et al., 2014), geographic regions (Pauw and Stanway, 2015), ecosystem type (Cirtwill et al., 2015) and in dependence on the specialization measure applied (Dalsgaard et al., 2017; Pinheiro et al., 2024).

Studies of the relationships between the degree of specialization and latitude have mostly been carried out for mutualistic networks involving free-living species, such as plant–insect pollinator (e.g. Ollerton and Cranmer, 2002), plant–hummingbird (e.g. Dalsgaard et al., 2011), tree–ant (Dáttilo and Vasconcelos, 2019) and bird–fruit plant (Ten Caten and Dallas, 2024) networks. Geographic patterns of specialization in antagonistic parasite–host networks have rarely been studied (but see Morris et al., 2014; de Angeli Dutra and Poulin, 2024; Pinheiro et al., 2024).

Furthermore, the majority of the latitudinal specialization gradient studies were carried out either for a restricted geographic span (e.g. Dáttilo and Vasconcelos, 2019; Pinheiro et al., 2024) or at the global scale (e.g. Morris et al., 2014; Ten Caten and Dallas, 2024). In the latter case, the real pattern can be masked by different patterns in different parts of the globe. Interaction patterns and, consequently, their spatial distribution can differ between biogeographic realms because of the interactors’ differential evolutionary histories and dispersal events. For example, Krasnov et al. (2024a) demonstrated that the latitudinal patterns of the species richness and phylogenetic and functional diversity of fleas and their mammalian hosts differ substantially between geographic quadrants.

A biogeographic realm is characterized by generally similar species compositions due to shared evolutionary histories shaped by geological division, climate and hard barriers such as oceans or mountain ridges. In other words, bipartite networks from different realms are composed of different species that likely respond differently to interacting partners, as well as to environmental factors, which, in turn, vary differently along latitudes. As a result, a latitudinal specialization gradient could be manifested differently in different realms. To the best of my knowledge, a comparison of latitudinal patterns of specialization between biogeographic realms has never been done, although biogeographic variation has been shown for other network properties (Guilhaumon et al., 2012; Krasnov et al., 2022, 2025; Krasnov and Shenbrot, 2025).

Here, I attempted to fill this gap by investigating the latitudinal patterns of phylogenetic, functional and numerical specialization in flea–mammal networks from 4 biogeographic realms. I adopted the terminology of Ten Caten and Dallas (2024) and termed the specialization of fleas and hosts in a network as interaction specificity. Interaction specificity is defined as the degree of phylogenetic or functional (i.e. trait-based) similarity between partners of a given species, that is, between fleas parasitic on a given host or hosts exploited by a given flea (Shefferson et al., 2019; Ten Caten and Dallas, 2024). Obviously, higher phylogenetic or functional similarity between partners indicates a species’ higher phylogenetic (PIS) or functional interaction specificity (FIS). In addition, interaction specificity can be expressed as the proportion of a species’ partners, relative to all potential partners in a network [(taxonomic interaction specificity sensu Ten Caten and Dallas, 2024)]. In application to parasites, the PIS, FIS and taxonomic interaction specificity facets reflect their host specificity in terms of phylogenetic relatedness, trait similarity and the size of their host spectra. This can also be applied to a set of parasites exploiting a given host, so that a host can be considered as a specialist or a generalist regarding the phylogenetic relatedness, functional similarity and relative species richness of its parasite assemblage (see Vázquez et al., 2005). Therefore, I chose to replace the term ‘taxonomic’ specificity (Ten Caten and Dallas, 2024) with numerical interaction specificity [NIS; analogous to the numerical host specificity of Rohde and Rohde (2008)]. According to the latitudinal specialization gradient pattern, the PIS, FIS and NIS facets are expected to decrease with an increase in absolute latitude.

Following Ten Caten and Dallas (2024), I considered interaction specificity at 3 organizational levels, namely intraspecific [= ‘population’ sensu Ten Caten and Dallas (2024)], interspecific [= ‘species’ sensu Ten Caten and Dallas (2024)] and community (Ten Caten and Dallas, 2024). This was done because the latitudinal pattern can be driven by different mechanisms at different organizational levels (Gaston et al., 2008; Ten Caten and Dallas, 2024). I asked whether the spatial patterns of interaction specificity followed the predictions of a latitudinal specialization gradient. I also asked whether these patterns (if any) differed between (a) fleas and hosts, (b) biogeographic realms, (c) facets of specialization (e.g. Krasnov et al., 2008; Krasnov and Shenbrot, 2025) and (d) organizational levels.

## Materials and methods

### Data on flea*–*mammal interaction networks

The data on the interactions between fleas and small mammalian hosts (Didelphimorphia, Macroscelidea, Afrosoricida, Scandentia, Eulipotyphla, Rodentia and the ochotonid Lagomorpha) for 15 Afrotropical regions, 23 Nearctic regions, 17 Neotropical regions and 36 Palearctic regions were taken from earlier studies (e.g. Krasnov et al., 2022). These data were compiled from various literature sources (including ‘grey’ publications) (see references and maps in, e.g., Krasnov et al., 2022). I focused on studies that aimed to compile the most complete lists of fleas on a given host species in a given region. In total, I used data on 868 host species infested by 972 flea species and demonstrating 12 205 unique interactions (Supplementary Material, Appendix 1, Table S1). Synanthropic ubiquitous host species (*Mus musculus, Rattus rattus* and *Rattus norvegicus*) and their characteristic fleas (*Leptopsylla segnis, Xenopsylla brasiliensis, Xenopsylla cheopis, Nosopsyllus fasciatus* and *Nosopsyllus londiniensis*) were not considered in the analyses. Data for each region were represented as a binary interaction matrix.

### Flea and host traits

Each flea species was characterized by 6 traits, namely (a) the number of host species exploited across a flea’s geographic range, (b) the phylogenetic diversity of these hosts, (c) ranked body size (small, medium or large), (d) the number of sclerotized ctenidia (no ctenidia, only a pronotal ctenidium, both pronotal and genal ctenidia), (e) microhabitat preference (preference to spend the most time in a host’s hair, its burrow/nest or no clear preference) and (f) the latitudinal span of its geographic range. Each host species was characterized by 5 traits known to be associated with variation in flea parasitism, namely (a) body mass, (b) relative brain mass, (c) dispersal range (the distance travelled by a species between the birth and the breeding location), (d) habitat breadth (the number of distinct level 1 IUCN habitats) and (e) geographic range size. These data were taken from earlier studies (Krasnov et al., 2016, 2024a; see rationale and references to the sources of information in these publications). Prior to analyses, the geographic range sizes of hosts were ln-transformed, and then, the continuous trait variables of both fleas and hosts were scaled from 0 to 1.

### Flea and host phylogenies

I constructed phylogenetic trees for fleas and hosts separately for each realm. As a backbone for flea phylogenies, I used the most comprehensive molecular phylogenetic tree of Zhu et al. (2015). This tree comprised the majority of flea genera, albeit no species, from the dataset used in this study. I established the topology of the remaining genera and species based on either their morphologically derived taxonomic positions (Hadfield *et al*., 2014) or molecular and morphological phylogenetic studies of several taxa (see references in Krasnov et al., 2022). No information on branch lengths was available. Therefore, I assigned all branches the length of 1, and arbitrarily ultrametrized the tree using the ‘force.ultrametric’ function (with the method = ‘extend’ option) of the ‘phytools’ package (Revell, 2012), implemented in the R Statistical Environment (R Core Team, 2025).

I took host phylogenetic trees (topology and branch lengths) as 1000 random subsets from the 10 000 species-level birth-death node-dated completed trees for the 5911 mammal species of Upham et al. (2019). I constructed consensus trees for each realm with the ‘consensus.edge’ function of the ‘phytools’ package. Then, I ultrametrized each resultant tree as described above and resolved polytomies using the ‘multi2di’ function of the R package ‘ape’ (Paradis and Schliep, 2019).

### Calculation of PIS, FIS and NIS

I calculated interaction specificity, following Ten Caten and Dallas (2024), using their R functions ‘phylogenetic_Specificity’, ‘functional_Specificity’ and ‘taxonomic_Specificity’ (for NIS) for each flea species and each host species in a region that interacted with at least 3 partners (otherwise, interaction specificity either cannot be calculated or would be highly unreliable). In particular, I calculated the PIS of a given flea or a host species as the mean pairwise phylogenetic distance (MPD) for all its interacting partners (i.e. all fleas parasitic on a given host or all hosts infected by a given flea, respectively). Estimation of PIS for a given species is strongly affected by the number of its interacting partners. Consequently, for each flea or host, I calculated the standardized MPD (*z*-score) computed as the difference between the observed MPD and the mean of 1000 null estimates of MPD (for randomly taken sets of the same number of partners from the same site) divided by the standard deviation of null estimates. This was done using the ‘ses.mpd’ function of the R package ‘picante’ (Kembel et al., 2010).

I calculated FIS as the mean pairwise functional dissimilarity of all partners of a given flea or host species. I computed functional dissimilarities between each pair of partners of a species from the distance trait matrix based on the Gower distance using the ‘compute_dist_matrix’ function of the R package ‘funrar’ (Grenié et al., 2017). The Gower dissimilarity coefficient allows constructing a dissimilarity matrix from data composed of different types of variables (quantitative, categorical, ordinal and binary) (Gower, 1971). Similarly to PIS, estimates of FIS are affected by the species richness of interaction partners. Consequently, I used the standardized mean functional specificity (*z*-score) calculated similarly to that of the standardized phylogenetic specificity.

NIS [‘taxonomic’ sensu Ten Caten and Dallas (2024)] was represented by the number of partners of a flea or a host species divided by the number of all potential partners in a given network. In other words, it is the proportion of the species in a community with which a given flea or a host species interacts.

### Data analysis

I tested for latitudinal patterns in PIS, FIS and NIS facets within each biogeographic realm separately for fleas and hosts. To avoid negative values of explanatory variable (i.e. latitude), I substituted the latitudinal coordinates of the centre of each region with its distance to the equator. I tested for latitudinal variation of interaction specialization at the intraspecific level in 2 ways. First, I considered the PIS, FIS and NIS facets for each flea and host species in each regional network and analysed the relationships between each interaction specificity facet and the distance of a network to the equator using phylogenetic linear mixed-effect models (PGLMM) with species identity as a random effect. This was done using the ‘pglmm’ function of the R package ‘phyr’ (Li et al., 2020) with *R^2^* values extracted using the R package ‘rr2’ (Ives and Li, 2018). Second, I selected flea and host species that occurred in at least 6 regional networks within a biogeographic realm (Supplementary Material, Appendix 1, Tables S2–S5) and tested for the relationships between each interaction specificity facet of each of these species and the distance of the centre of the region where it occurred to the equator using ordinary least squares (OLS) linear models.

At the interspecific level, I averaged each value of a given interaction specificity facet for each flea and host species across all networks where this species occurred within a realm. Then, I applied phylogenetic generalized least squares models, using the R package ‘caper’ (Orme et al., 2025), to test for the pattern of interaction specificity variation along the gradient of average distance to the equator of the centres of the regions in which a species occurred.

At the community level, I averaged the values of each interaction specificity facet across all species of a given interaction group (i.e. fleas and hosts) in a given regional network. Then, I used the OLS linear models to test for the relationship between the PIS, FIS or NIS of a regional network and the distance of the centre of the region to the equator.

I intentionally did not apply the adjustments of the alpha-level for multiple comparisons. These procedures (e.g. Bonferroni corrections) have been strongly criticized by both statisticians and ecologists because of an inflated rate of Type II errors (e.g. Rothman, 1990; Perneger, 1998; Nakagawa, 2004).

## Results

### Intraspecific level

**Figure 1.**
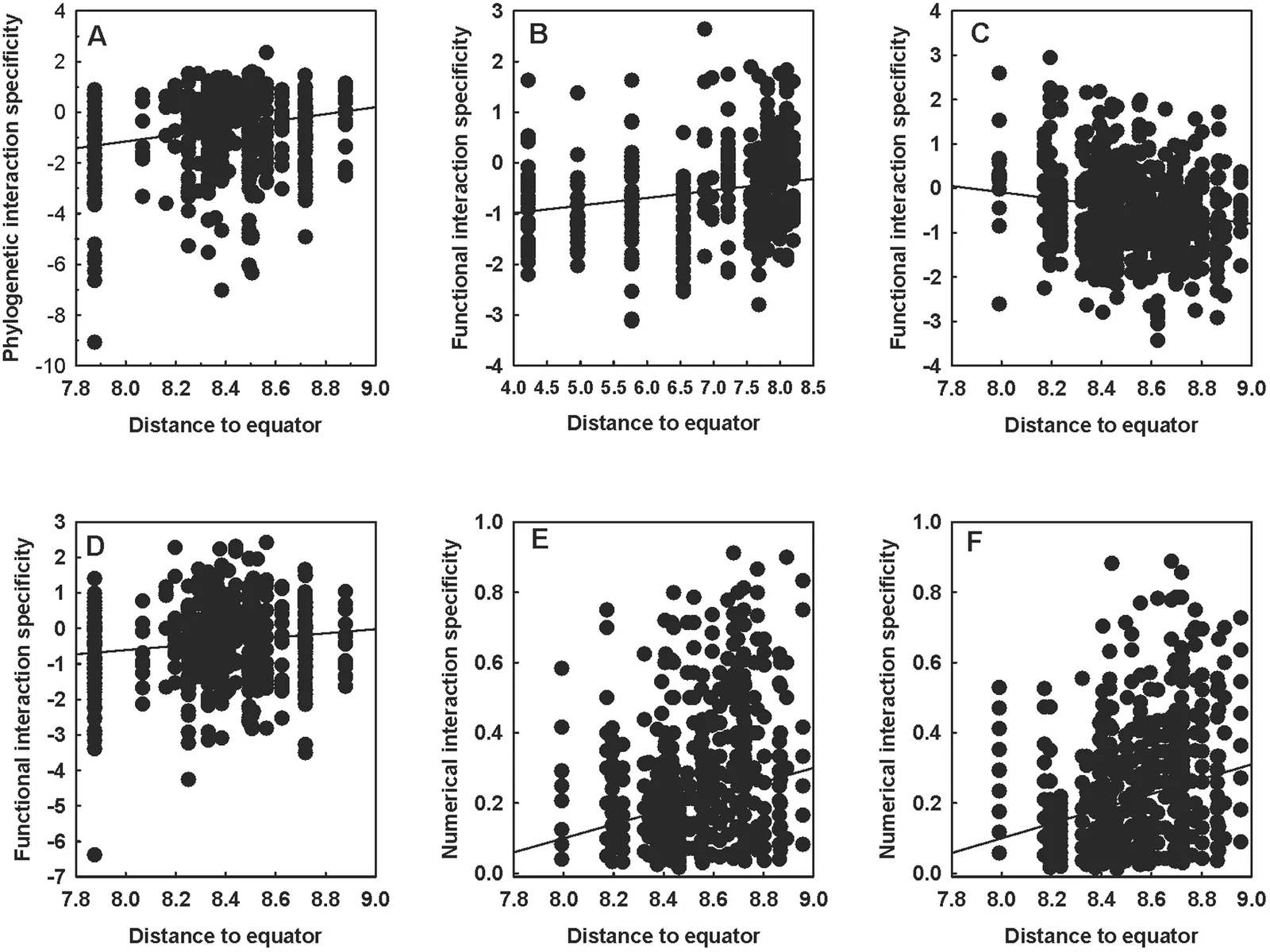
Relationships between the distance of a flea–mammal network to the equator (ln-transformed) and (A) the phylogenetic interaction specificity of hosts in the Nearctic, (B) the functional interaction specificity of fleas in the Afrotropics, (C) the functional interaction specificity of fleas in the Palearctic, (D) the functional interaction specificity of hosts in the Nearctic, (E) the numerical interaction specificity of fleas in the Palearctic and (F) the numerical interaction specificity of hosts in the Palearctic at the intraspecific level. Phylogenetic and functional specificity facets are calculated as *z*-scores from null models (see text for explanation). Figure 1 long description.

At the intraspecific level, significant relationships between PIS and the distance of a network to the equator were detected only in the Afrotropical and the Nearctic hosts (positive in both realms) (Table 1, see example in Fig. 1A). The FIS of fleas varied with latitude in all realms, either increasing (in the Afrotropics and the Neotropics) or decreasing (in the Nearctic and the Palearctic) with the distance of a network to the equator (Table 1; see examples in Fig. 1B and 1C). In hosts, a significant relationship between FIS and distance to the equator (positive) was detected in the Nearctic only (Table 1, Fig. 1D). The numerical host specificity of both fleas and hosts increased with the distance to the equator in 3 of the 4 realms (except the Neotropics) (Table 1; see examples in Fig.f 1E and 1F).

**Table 1.** Relationships between the phylogenetic (Phy), functional (Fun) and numerical (Num) interaction specificity facets of fleas and hosts in flea–host interaction networks from 4 biogeographic realms and the distance of a network to the equator (DE, ln-transformed) at the intraspecific level Table 1 long description.

Realm	Group	Equation	*T*	*P*	*R*^2^
Afrotropics	Fleas	Fun = −1·59 + 0·15 × DE	3·59	<0·001	0·09
		Num = 0·02 × DE	4·14	<0·001	0·37
	Hosts	Phy = −2·13 + 0·21 × DE	3·85	<0·001	0·23
		Num = 0·03 × DE	6·16	<0·001	0·32
Nearctic	Fleas	Fun = 2·44–0·32 × DE	−2·32	0·02	0·06
		Num = −0·26 + 0·04 × DE	3·18	<0·001	0·15
	Hosts	Phy = −11·95 + 1·35 × DE	4·32	<0·001	0·25
		Fun = −5·33 + 0·59 × DE	2·63	0·01	0·19
		Num = −0·37 + 0·05 × DE	3·14	0·01	0·25
Neotropics	Fleas	Fun = −1·86 + 0·22 × DE	3·36	<0·001	0·06
Palearctic	Fleas	Fun = 5·59–0·71 × DE	−3·77	<0·001	0·08
		Num = −1·51 + 0·20 × DE	6·67	<0·001	0·21
	Hosts	Num = −1·58 + 0·21 × DE	6·25	<0·001	0·28

Only models with significant (*P* < 0·05) slopes are shown.

**Figure 2.**
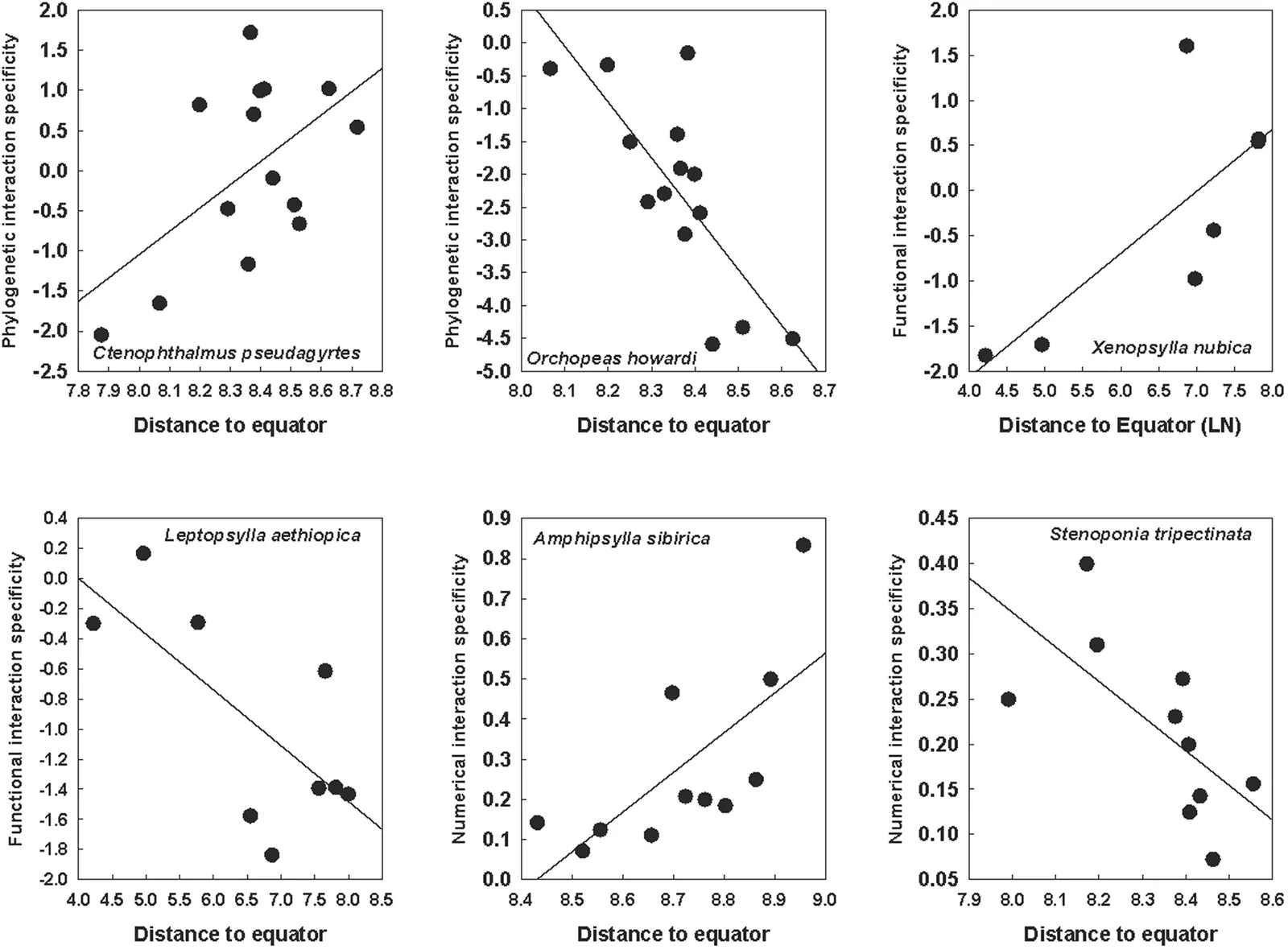
Relationships between the distance of a network to the equator and phylogenetic (for the Nearctic fleas *Ctenophthalmus pseudagyrtes* and *Orchopeas howardi*), functional (for the Afrotropical fleas *Xenopsylla nubica* and *Leptopsylla aethiopica*) and numerical (for the Palearctic fleas *Amphipsylla sibirica* and *Stenoponia tripectinata*) interaction specificity facets in flea–mammal networks at the intraspecific level. Phylogenetic and functional specificity facets are calculated as *z*-scores from null models (see text for explanation). Figure 2 long description.

**Figure 3.**
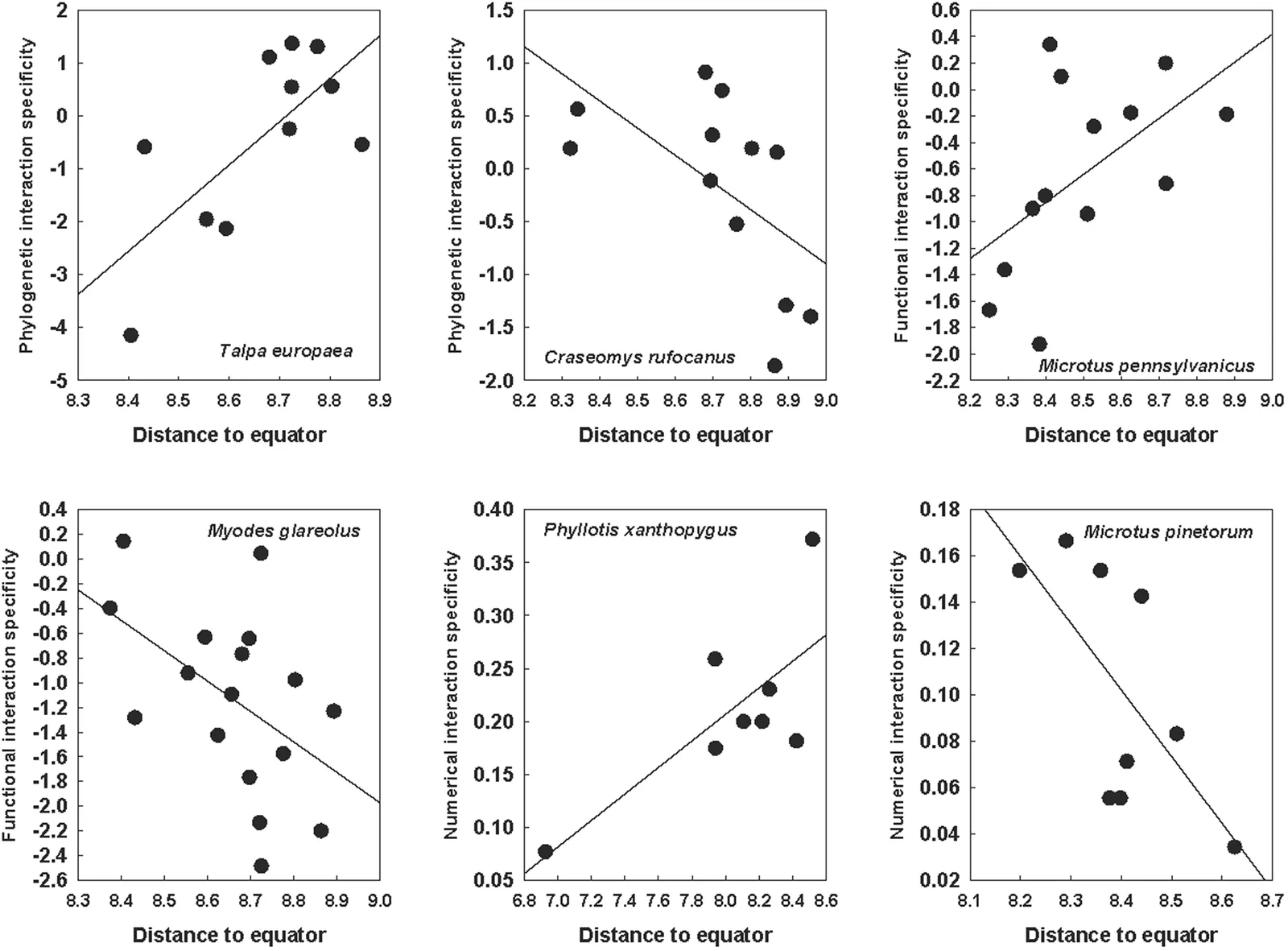
Relationships between the distance of a network to the equator and phylogenetic (for the Palearctic hosts *Talpa europea* and *Craseomys rufocanus*), functional (for the Nearctic host *Microtus pennsylvanicus* and the Palearctic host *Myodes glareolus*) and numerical (for the Neotropical host *Phyllotis xanthopygus* and the Nearctic host *Microtus pinetorum*) interaction specificity facets in flea–mammal networks at the intraspecific level. Phylogenetic and functional specificity facets are calculated as *z*-scores from null models (see text for explanation). Figure 3 long description.

These trends were revealed when the latitudinal patterns of interaction specificity were considered across all flea or host species. However, variable trends were detected when the relationships between interaction specificity and the distance of a network to the equator were tested for species that occurred in at least 6 networks. PIS, FIS and NIS increased with distance to the equator in some species and decreased with this distance in other species (Tables 2 and 3; see examples in Fig. 2 for fleas and Fig. 3 for hosts).

**Table 2.** Relationships between the phylogenetic (Phy), functional (Fun) and numerical (Num) interaction specificity facets of fleas occurring in at least 6 flea–host interaction networks from 4 biogeographic realms and the distance of a network to the equator (DE, ln-transformed) at the intraspecific level Table 2 long description.

Realm	Species	Equation	*t*	*R*^2^
Afrotropics	*Leptopsylla aethiopica*	Phy = 9·20 + 1·28 × DE	3·65*	0·66
	*Leptopsylla aethiopica*	Fun = 5·3–1·25 × DE	−2·65*	0·50
	*Xenopsylla nubica*	Fun = 6·81 + 0·83 × DE	2·57*	0·57
	*Ctenophthalmus calceatus*	Fun = 3·93 + 10·56 × DE	2·73*	0·52
	*Chiastopsylla rossi*	Num = 6·50 + 2·55 × DE	6·29***	0·89
Nearctic	*Ctenophthalmus pseudagyrtes*	Phy = 8·37 + 0·11 × DE	2·32*	0·31
	*Orchopeas howardi*	Phy = 8·20–0·07 × DE	−4·20***	0·60
	*Ctenophthalmus pseudagyrtes*	Fun = 8·53 + 0·12 × DE	2·20*	0·29
	*Orchopeas caedens*	Fun = 8·36–0·22 × DE	−3·43**	0·63
	*Peromyscopsylla scotti*	Num = 8·51 – 0·91 × DE	−3·31**	0·61
	*Opisodasys keeni*	Num = 8·23 + 3·38 × DE	4·21**	0·75
Neotropics	*Polygenis atopus*	Num = 6·51–·71 × DE	3·64**	0·69
Palearctic	*Amphipsylla sibirica*	Phy = 8·87 + 0·06 × DE	2·26*	0·39
	*Amphipsylla rossica*	Fun = 8·50–0·09 × DE	−2·48*	0·32
	*Ctenophthalmus assimilis*	Fun = 8·74 + 0·06 × DE	2·46*	0·36
	*Stenoponia tripectinata*	Fun = 8·25–0·13 × DE	−2·39*	0·42
	*Amphipsylla sibirica*	Num = 8·57 + 0·51 × DE	3·01**	0·5
	*Leptopsylla taschenbergi*	Num = 8·20 + 1·12 × DE	2·29*	0·4
	*Peromyscopsylla silvatica*	Num = 8·56–0·50 × DE	2·54*	0·45
	*Stenoponia tripectinata*	Num = 8·58–1·14 × DE	−2·49*	0·44

Only species for which the models demonstrated significant (*P* < 0·05) slopes are shown.

* *P* < 0·05, **P < 0·01, ***P < 0·001.

**Table 3.** Relationships between the phylogenetic (Phy), functional (Fun) and numerical (Num) interaction specificity facets of hosts occurring in at least 6 flea–host interaction networks from 4 biogeographic realms and the distance of a network to the equator (DE, ln-transformed) at the intraspecific level Table 3 long description.

Realm	Species	Equation	*t*	*R*^2^
Afrotropics	*Gerbilliscus brantsii*	Phy = 7·84 + 0·15 × DE	2·90*	0·63
	*Mus minutoides*	Phy = 7·51 + 0·32 × DE	6·53***	0·90
	*Gerbilliscus brantsii*	Fun = 7·84 + 0·23 × DE	4·70**	0·82
	*Aethomys namaquensis*	Num = 6·89 + 1·94 × DE	3·04*	0·61
	*Gerbilliscus brantsii*	Num = 5·24 + 5·40 × DE	3·14*	0 ·62
	*Gerbilliscus leucogaster*	Num = 5·56 + 4·46 × DE	2·67*	0 ·50
	*Rhabdomys pumilio*	Num = 5·73 + 4·92 × DE	2·91*	0·46
Nearctic	*Peromyscus leucopus*	Phy = 8·37 + 0·13 × DE	3·10**	0·45
	*Tamias minimus*	Phy = 8·54–0·10 × DE	−2·56*	0·57
	*Microtus pennsylvanicus*	Fun = 8·59 + 0·14 × DE	2·18*	0·3
	*Microtus pennsylvanicus*	Num = 8·27 + 1·02 × DE	3·11**	0·45
	*Microtus pinetorum*	Num = 8·56–1·61 × DE	−2·46*	0·46
	*Tamias minimus*	Num = 8·32 + 2·69 × DE	2·48*	0·47
Neotropics	*Oligoryzomys longicaudatus*	Num = 7·24 + 4·21 × DE	2·84*	0·62
	*Philander opossum*	Num = 5·87 + 4·39 × DE	2·82*	0·67
	*Phyllotis xanthopygus*	Num = 7·11 + 4·38 × DE	2·71*	0·55
Palearctic	*Craseomys rufocanus*	Phy = 8·69–0·13 × DE	−2·21*	0·33
	*Microtus oeconomus*	Phy = 8·75–0·03 × DE	−2·13*	0·27
	*Talpa europaea*	Phy = 8·69 + 0·06 × DE	−2·16*	0·30
	*Myodes glareolus*	Fun = 8·55–0·10 × DE	2·96*	0·49
	*Myodes rutilus*	Fun = 8·66–0·08 × DE	−2·20*	0·24
	*Myodes rutilus*	Num = 8·46 + 0·51 × DE	3·24**	0·47
	*Sciurus vulgaris*	Num = 8·50 + 1·11 × DE	2·12*	0·26

Only species for which the models demonstrated significant (*P* < 0·05) slopes are shown.

* *P* < 0·05, **P < 0·01, ***P < 0·001.

### Interspecific level

**Figure 4.**
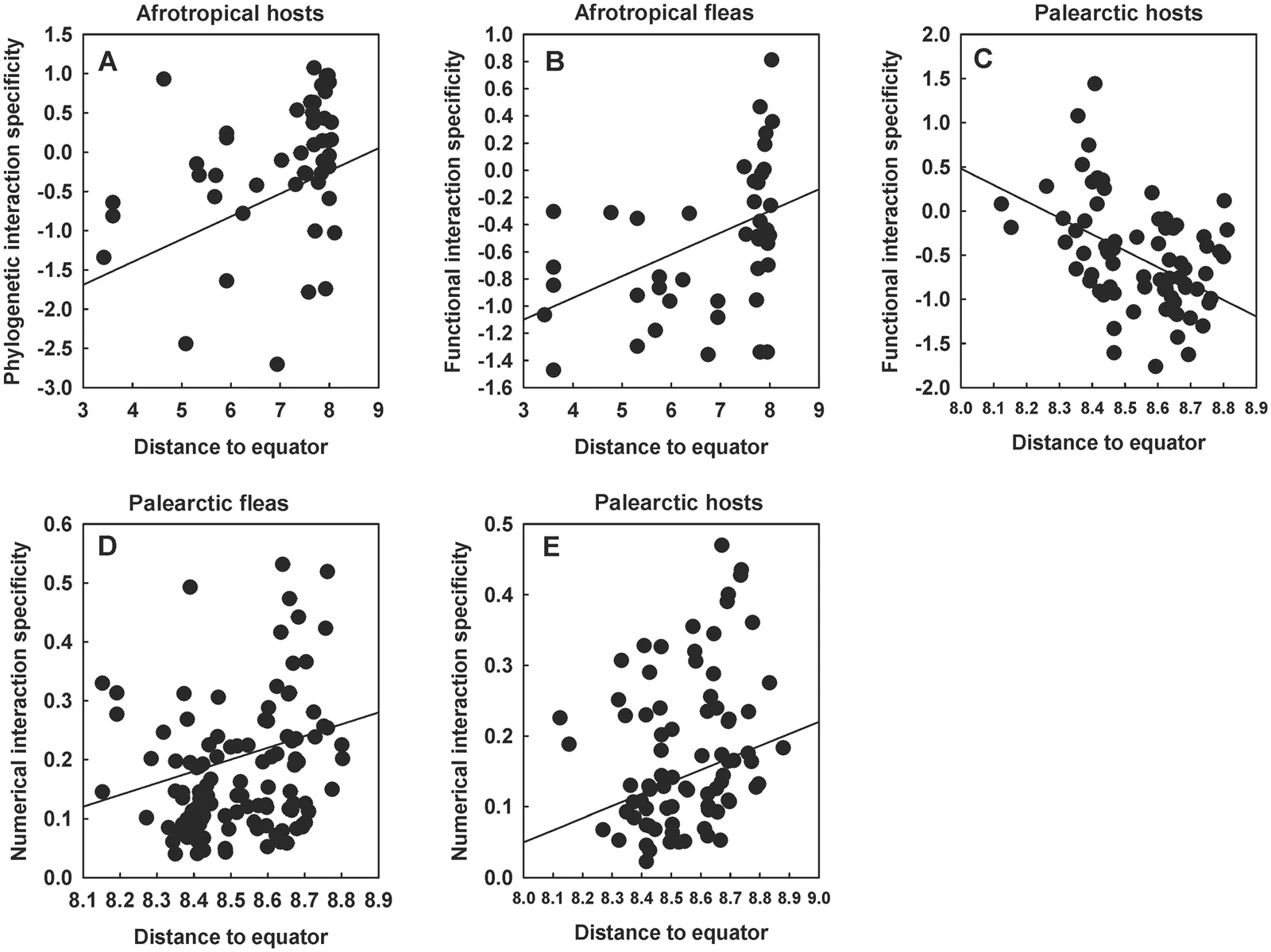
Relationships between the distance of a flea–mammal network to the equator (ln-transformed) and (A) the phylogenetic interaction specificity of hosts in the Afrotropics, (B) the functional interaction specificity of fleas in the Afrotropics, (C) the functional interaction specificity of hosts in the Palearctic, (D) the numerical interaction specificity of fleas in the Palearctic and (E) the numerical interaction specificity of hosts in the Palearctic at the interspecific level. Phylogenetic and functional specificity facets are calculated as *z*-scores from null models (see text for explanation). Figure 4 long description.

Across species, significant latitudinal patterns of interaction specificity were found in the Old World but not the New World realms. Specifically, PIS increased farther from the equator in hosts but not in fleas and in the Afrotropics but not in the Palearctic (Table 4, see example in Fig. 4A). Relationships between the FIS of fleas and the distance of a network to the equator were detected in both realms, but the trends were opposite, namely positive in the Afrotropics and negative in the Palearctic (Table 4, see examples in Fig. 4B and 4C, respectively). No association between this interaction specificity facet at this organizational level and latitude was found for hosts in either realm. Both fleas and hosts in both realms demonstrated significantly lower NIS (i.e. normalized fraction of interacting partners) in the networks relatively closer to the equator (Table 4, see examples in Fig. 4D for fleas and Fig. 4E for hosts).

**Table 4.** Relationships between the phylogenetic (Phy), functional (Fun) and numerical (Num) interaction specificity facets of fleas and hosts in flea–host interaction networks from 4 biogeographic realms and the distance of a network to the equator (DE, ln-transformed) at the interspecific level Table 4 long description.

Realm	Group	Equation	*t*	*P*	*R*^2^
Afrotropics	Fleas	Phy = −2·56 + 0·29 × DE	3·44	0·001	0·23
		Num = 0·02 × DE	2·30	0·02	0·32
	Hosts	Phy = −2·56 + 0·29 × DE	3·44	0·001	0·23
		Num = 0·02 × DE	2·30	0·02	0·32
Palearctic	Fleas	Fun = 15·36–1·86 × DE	−4·40	<0·001	0·08
		Num = −1·50 + 0·20 × DE	2·86	0·01	0·21
	Hosts	Num = −1·31 + 0·17 × DE	2·33	0·02	0·28

Only models with significant (*P* < 0·05) slopes are shown.

### Community level

**Figure 5.**
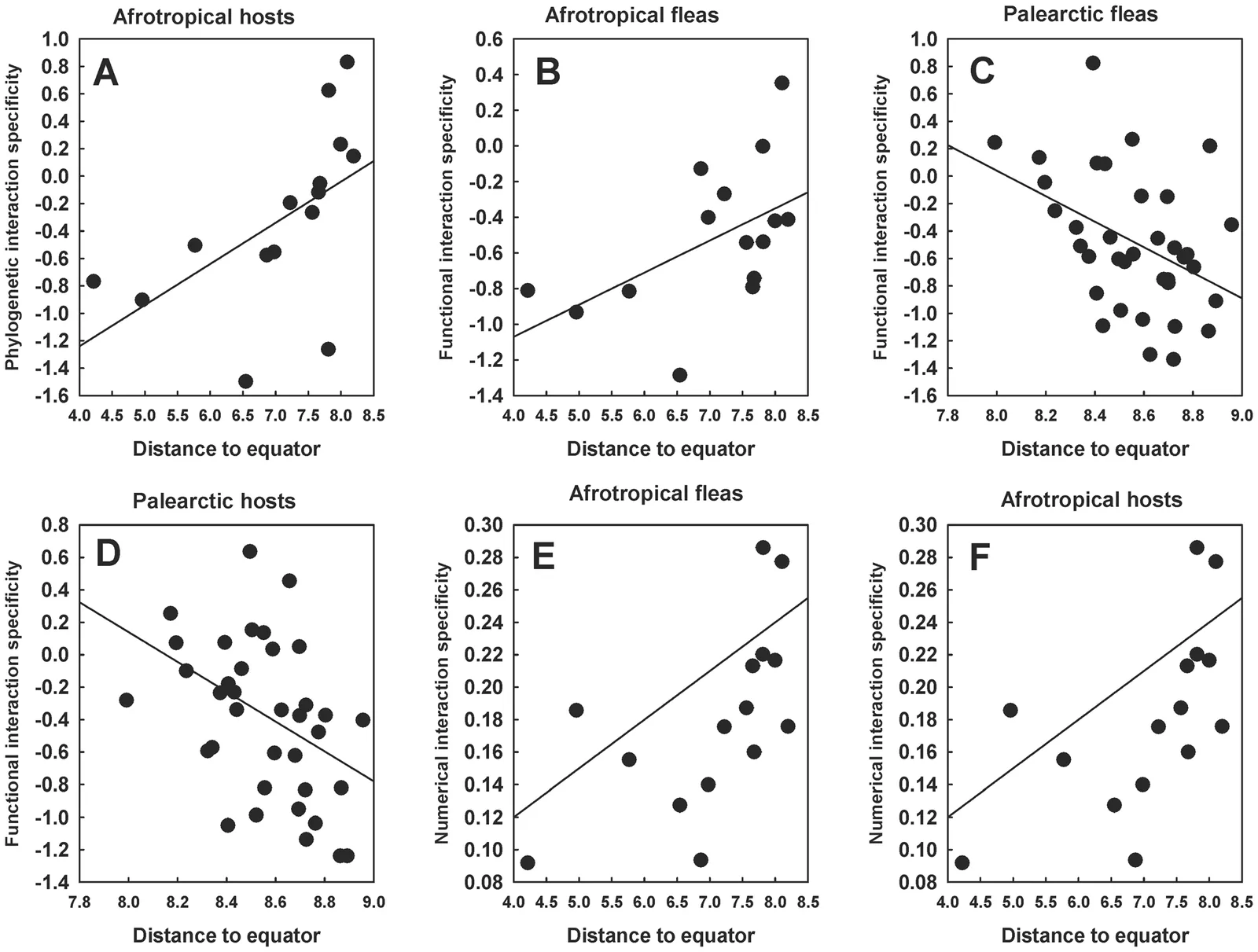
Relationships between the distance of a flea–mammal network to the equator (ln-transformed) and (A) the phylogenetic interaction specificity of hosts in the Afrotropics, (B) the functional interaction specificity of fleas in the Afrotropics, (C) the functional interaction specificity of fleas in the Palearctic, (D) the functional interaction specificity of hosts in the Palearctic, (E) the numerical interaction specificity of fleas in the Afrotropics and (F) the numerical interaction specificity of hosts in the Afrotropics at the community level. Phylogenetic and functional specificity facets are calculated as *z*-scores from null models (see text for explanation). Figure 5 long description.

Similarly to the interspecific level, associations between the 3 interaction specificity facets and latitude at the community level were found in the Old World realms only. Again, the PIS of hosts, but not fleas, and in the Afrotropics, but not the Palearctic, demonstrated latitudinal trends (Table 5, Fig. 5A). Latitudinal trends of flea FIS were revealed in both the Afrotropics and the Palearctic (positive in the former and negative in the latter realm; Table 5, Fig. 5C and 5D). Positive relationships between NIS consistently increased with an increase in the distance to the equator in both interactors in both these realms (Table 5, see examples in Fig. 5E and 5F).

**Table 5.** Relationships between the functional (Fun) and numerical (Num) interaction specificity facets of fleas and hosts in flea–host interaction networks from 4 biogeographic realms and the distance of a network to the equator (DE, ln-transformed) at the community level Table 5 long description.

Realm	Group	Equation	*t*	*P*	*R*^2^
Afrotropics	Fleas	Fun = −1·79 + 0·18 × DE	2·26	0·04	0·09
		Num = 0·03 × DE	2·96	0·02	0·39
	Hosts	Fun = −2·44 + 0·30 × DE	2·41	0·03	0·22
		Num = 0·03 × DE	2·69	0·02	0·32
Palearctic	Fleas	Fun = 7·48–0·93 × DE	−2·69	0·01	0·08
		Num = −1·69 + 0·23 × DE	3·21	0·001	0·21
	Hosts	Fun = 7·50–0·92 × DE	−2·76	0·01	0·10
		Num = −1·69 + 0·23 × DE	3·21	<0·001	0·28

Only models with significant (*P* < 0·05) slopes are shown.

## Discussion

The manifestation of the relationships between interaction specificity in a network and the distance of this network to the equator (a proxy for latitude) differed between (a) interactor groups, (b) specificity facets, (c) organizational levels and (b) biogeographic realms. Moreover, when a latitudinal pattern of interaction specificity was found, it conformed to the latitudinal specialization gradient in some cases but opposed it in other cases.

The results of this study demonstrated that spatial patterns appeared to be more characteristic of FIS in fleas and of PIS in hosts. In addition, the direction of the relationship between the values of interaction specificity and the distance to the equator was either positive or negative in fleas but was consistently positive in hosts (except for the FIS at the community level in the Palearctic). Further, I will discuss the results of this study concerning fleas in more detail than those concerning hosts. This is due to the asymmetry in parasite and host roles in their interactions, with fleas being the ‘active’ partner in the interaction networks. Although hosts are not merely submissive victims of fleas and they do defend themselves against flea parasitism using various tools (see Krasnov, 2008 for review), it is fleas that select their hosts (often actively and based on fitness-related decisions; Krasnov et al., 2004) and not vice versa.

Although some flea species demonstrated a latitudinal pattern of PIS (Table 2), this was not further translated into a general trend at the intraspecific, interspecific or community levels. The likely reasons behind this were that (a) the latitudinal pattern of phylogenetic specificity varied between different species, being either positive (e.g. the Nearctic *Ctenophthalmus pseudagyrtes*, Table 2) or negative (e.g. the Nearctic *Orchopeas howardi*, Table 2) or non-existent (e.g. the Nearctic *Aetheca wagneri*, Supplementary Material, Appendix 1, Table S3), and (b) the number of flea species that choose a host because of its phylogenetic position could be generally low. This suggests that the majority of fleas locally select their hosts based mainly on their traits (Krasnov et al., 2016), with these traits being shared between hosts regardless of these hosts’ phylogenetic affinities. The direction of the latitudinal pattern of flea PIS was similar at all organizational levels within a realm but not between realms. In particular, the hosts of Afrotropical fleas in the farther-from-the-equator networks were more similar to each other in their traits than hosts in the closer-to-the-equator networks. However, the opposite was the case in the Palearctic fleas. In other words, the Palearctic fleas demonstrated a latitudinal specialization gradient, whereas the Afrotropical fleas showed the inverse pattern. The NIS of fleas consistently increased with the distance of a network to the equator, indicating that the fleas from temperate zones used a higher proportion of available hosts than the tropical fleas, so that the latter appeared to be more specialized than the former, thus conforming to a latitudinal specialization gradient.

Intraspecifically, a latitudinal pattern of flea interaction specificity was detected in all realms, except numerical specificity in the Neotropics, while FIS varied with latitude across species and communities in the Old World, but not the New World, realms. The reason behind the lack of the latitudinal pattern of functional and numerical specificity at the interspecific and community levels in the Nearctic and the Neotropics could be that the interaction specificity of some species responded positively to environmental conditions associated with the latitude, whereas the specificity of some species responded negatively to these conditions, while the specificity of other species was latitude-invariant. For example, the functional specificity of the Nearctic *Ctenophthalmus pseudagyrtes* and *Orchopeas caedens* demonstrated opposite relationships with latitude (Table 2), whereas no association between functional specificity and latitude was detected in *Epitedia wenmanni*. This explanation, however, is substantially weakened by the fact that similar between-species variation, in response to latitude, was characteristic for the Afrotropical and the Palearctic fleas (Table 2), but their functional specificity at the interspecific and community levels was characterized by latitudinal patterns. Perhaps, the occurrence of latitudinal patterns at higher organizational levels is determined by the mere proportion of species demonstrating this pattern at the lowest organizational level. A higher proportion of species sensitive to latitudinal environmental variation would result in a higher probability of this variation to be manifested at higher levels.

A latitudinal specialization gradient is explained by (a) the higher species richness in tropical communities than in temperate and polar zones, and the subsequent fine-grained niche partitioning (MacArthur, 1969, 1984), and (b) the lower resource availability in temperate and polar zones than in the tropics (Pianka, 1966). As a result of the latter, consumer species in temperate or polar zones are forced to select a relatively high portion of the limited spectrum of resource species. Regarding fleas, the spectrum of their resource species (i.e. hosts) is determined not only by the species richness of these hosts but also by the number of hosts suitable for successful flea reproduction. In the majority of flea species and in almost all species exploiting small mammals, pre-imaginal development occurs completely off-host in hosts’ burrows (Marshall, 1981; Krasnov, 2008). Furthermore, flea eggs and larvae are highly sensitive to air temperature and relative humidity (e.g. Krasnov et al., 2001; Kreppel et al., 2016). Therefore, many fleas prefer ground-dwelling hosts possessing burrows with stable microclimatic conditions (Kucheruk, 1983; Shenbrot et al., 2002) over arboreal hosts with shelters exposed to external conditions (e.g. Havera, 1979). In general, the species richness of burrowing small mammals is higher in temperate zones than in the tropics (Pinkert et al., 2025). In addition, the depth and, consequently, the microclimatic stability of small mammals’ burrows increase in colder climates (Kucheruk, 1983). In other words, the availability and diversity of resources (i.e. hosts) for fleas could be lower in temperate zones than in tropical zones, but the opposite could be true for the suitability of these resources. Therefore, the higher availability of hosts possessing suitable shelters at higher latitudes may be the reason for interaction specificity decreasing towards the equator. However, this pattern was found for fleas in the Northern Hemisphere (the Nearctic and the Palearctic), while the opposite was the case for the Southern Hemisphere (the Afrotropics and the Neotropics). The main difference between the southern and the northern realms is that the actual tropics occur in the former but not in the latter. One possible explanation for the negative relationships between the distance to the equator and the FIS and NIS of fleas in the southern realms could be the generally higher species richness of hosts in African and South American tropical zones, which may promote the evolutionary development of flea traits that would facilitate the exploitation of multiple host species (Guimarães et al., 2011; Ten Caten and Dallas, 2024; but see Barabás et al., 2022).

The question of the geographic variation in the PIS, FIS and NIS of hosts in flea–host networks is essentially a question of the geographic variation in the PIS, FIS and NIS of flea assemblages harboured by a given host species. It was previously found that the higher latitudes in the Palearctic were associated with a higher degree of phylogenetic clustering (i.e. higher PIS) of the fleas harboured, albeit in only 4 of the 12 studied host species (Krasnov et al., 2013). In the current study, I did not find any latitudinal pattern of host phylogenetic specificity in the Palearctic but detected it in the Nearctic and the Afrotropics. It is possible that the mechanism behind the lack of a latitudinal trend in host phylogenetic specificity in the Palearctic and the Neotropics is similar to that implied for the explanation of the lack of a latitudinal pattern of flea functional and numerical specificity at the interspecific and community levels in the Nearctic and the Neotropics. Different hosts might demonstrate either opposite or no relationships between phylogenetic specificity and distance to the equator. The result would be the lack of a general trend. Between-host variation in the response of phylogenetic specificity to latitude could also be the case for the Afrotropics and the Nearctic, but the relative numbers of species with either negative or positive or no latitudinal pattern of phylogenetic specificity could result in either the occurrence of a general phylogenetic specificity–latitude trend or not. I recognize that this explanation is highly speculative and warrants further investigation.

From the functional perspective, the diversity of flea assemblages harboured by a host (i.e. its FIS) varied with latitude only in the Nearctic and Palearctic but in opposite directions. This difference may be because the latitudinal trend was detected at the intraspecific level in the Nearctic and at the community level in the Palearctic. As mentioned above, interaction specificity is driven by different mechanisms at different organizational levels (Ten Caten and Dallas, 2024). It is possible that the functional responses of the Nearctic populations of different species to environmental conditions along latitudes are similar, but the high variation in the strength of these responses masks latitudinal trends when these responses are averaged within and between species. In the Palearctic, the latitudinal trend in functional specificity was only revealed when averaged across all species because the responses of populations of a given species (i.e. at the intraspecific level) and the responses averaged within a species (i.e. at the interspecific level) were either too weak or too variable. These discrepancies could arise due to the high functional similarity of flea assemblages that are characteristic only for hosts possessing certain sets of traits (see examples in Krasnov et al., 2024b).

The latitudinal patterns of host NIS (i.e. the relative species richness of flea assemblages) were similar to those of fleas, being higher at higher absolute latitudes, thus supporting the expectations from the latitudinal specialization gradient hypothesis. Similarly to fleas, this was mainly characteristic for the Old World and not the New World realms. This again suggests that macroecological patterns, especially those concerning parasite–host interactions, may be realm-specific.

The between-realm differences in the manifestation of latitudinal patterns of interaction specificity in both fleas and hosts most likely result from (a) the different evolutionary histories of and (b) the landscape effects on flea–host interactions. The ancestral geographic regions of fleas are thought to be Australia and South America, connected via Antarctica (Zhu et al., 2015). Following dispersal of their hosts, fleas migrated from South to North America and then to the Old World, with some of them later returning (Zhu et al., 2015). As a result, the modern flea faunas of the Neotropics and the Nearctic represent a mix of ancient and relatively young taxa (Medvedev, 2005). Multiple series of host and subsequent flea migrations caused different flea families and genera to be predominant in different biogeographic realms (Medvedev, 2005). As a result, the structural properties of flea–host interaction networks differ between realms (e.g. Krasnov et al., 2022). In addition, biogeographic realms differ in the occurrence and spatial directions of hard geographic barriers, such as mountain ridges, which stretch along main longitudinal directions in the New World realms and latitudinal directions in the Old World realms. This difference can also result in the differences in flea and host dispersal patterns and, ultimately, flea–host interactions.

In conclusion, I found evidence of a latitudinal specialization gradient in some, but not other, biogeographic realms. This suggests that the occurrence and direction of the latitudinal specialization gradient are associated with a combination of evolutionary, historical and ecological factors. Moreover, when a macroecological pattern is considered at the global scale, a strong trend occurring in 1 biogeographic realm may mask a weak or an absent trend in another biogeographic realm. This may result in the absence of any trend at the global scale. Consequently, pooling data from regions with different biogeographical histories and landscape structures for a macroecological analysis may produce spurious results.

## Supporting information

10.1017/S0031182026102182.sm001 Krasnov supplementary material Krasnov supplementary material

## Data Availability

Raw data on flea and host species at the scale of biogeographic realms are contained in the sources cited in Krasnov *et al.* (2022) and in the Mendeley Data repository (Krasnov, 2025).

## Supplementary material

The supplementary material for this article can be found at https://doi.org/10.1017/S0031182026102182.

## Figure 1 Long description

The image consists of six scatter plots labeled A to F, each depicting different interaction specificity measures against the distance to the equator. In plot A, the x-axis represents Distance to equator ranging from 7.8 to 9.0 and the y-axis represents Phylogenetic interaction specificity ranging from negative 4 to positive 4. The plot shows a weak positive trend with points scattered throughout and no notable clusters or outliers. Plot B has the x-axis labeled Distance to equator from 4.0 to 8.5 and the y-axis labeled Functional interaction specificity from negative 4 to positive 4. A weak positive trend is visible, with points densely packed around the middle range. Plot C shows Distance to equator from 7.8 to 9.0 on the x-axis and Functional interaction specificity from negative 4 to positive 4 on the y-axis. A weak negative trend is observed, with points spread evenly. Plot D features Distance to equator from 7.8 to 9.0 on the x-axis and Functional interaction specificity from negative 4 to positive 4 on the y-axis. The plot indicates a weak positive trend, with points densely clustered in the middle. Plot E has Distance to equator from 7.8 to 9.0 on the x-axis and Numerical interaction specificity from 0.0 to 1.0 on the y-axis. A positive trend is evident, with points concentrated at higher values. Plot F shows Distance to equator from 7.8 to 9.0 on the x-axis and Numerical interaction specificity from 0.0 to 1.0 on the y-axis. A positive trend is visible, with points densely packed at higher values. All plots use the same symbol for data points and no color coding or legend is present.

Navigate back to Figure 1.

## Figure 2 Long description

The image contains six scatter plots, each representing a different flea species and interaction specificity facet in relation to distance from the equator. 1. Ctenophthalmus pseudagyrtes: The x-axis is labeled Distance to equator, ranging from 7.3 to 8.8. The y-axis is Phylogenetic interaction specificity, ranging from negative 2.5 to 2.0. The plot shows an upward trend with a fitted line, indicating a positive relationship. 2. Orchopeas howardi: The x-axis is Distance to equator, ranging from 8.0 to 8.7. The y-axis is Phylogenetic interaction specificity, ranging from negative 5.0 to 0.5. The plot shows a downward trend with a fitted line, indicating a negative relationship. 3. Xenopsylla nubica: The x-axis is Distance to Equator (LN), ranging from 4.0 to 7.5. The y-axis is Functional interaction specificity, ranging from negative 2.0 to 2.0. The plot shows an upward trend with a fitted line, indicating a positive relationship. 4. Leptopsylla aethiopica: The x-axis is Distance to equator, ranging from 4.0 to 8.5. The y-axis is Functional interaction specificity, ranging from negative 2.0 to 0.2. The plot shows a downward trend with a fitted line, indicating a negative relationship. 5. Amphipsylla sibirica: The x-axis is Distance to equator, ranging from 8.4 to 9.0. The y-axis is Numerical interaction specificity, ranging from 0.0 to 0.9. The plot shows an upward trend with a fitted line, indicating a positive relationship. 6. Stenoponia tripectinata: The x-axis is Distance to equator, ranging from 7.9 to 8.6. The y-axis is Numerical interaction specificity, ranging from 0.05 to 0.45. The plot shows a downward trend with a fitted line, indicating a negative relationship. Across the panels, species show varying relationships with distance to the equator, with some increasing and others decreasing in interaction specificity. The plots use point markers and fitted lines to visually encode data, highlighting trends and deviations. The overall purpose is to illustrate how interaction specificity facets change with geographical distance for different flea species.

Navigate back to Figure 2.

## Figure 3 Long description

The image contains six scatter plots, each showing interaction specificity facets against distance to the equator for different species. The horizontal axis in all plots is labeled 'Distance to equator' with values ranging from approximately 6.8 to 9.0. The vertical axis varies per plot, representing phylogenetic, functional, or numerical interaction specificity. 1. Talpa europaea: The plot shows phylogenetic interaction specificity with a positive trend. Points are scattered, with a fitted regression line indicating an increase in specificity as distance increases. 2. Craseomys rufocanus: This plot also represents phylogenetic interaction specificity, showing a negative trend. The regression line slopes downward, indicating a decrease in specificity with increasing distance. 3. Microtus pennsylvanicus: Functional interaction specificity is plotted, showing a positive trend. The regression line slopes upward, suggesting increased specificity with distance. 4. Myodes glareolus: The plot shows functional interaction specificity with a negative trend. The regression line slopes downward, indicating decreased specificity with distance. 5. Phyllotis xanthopygus: Numerical interaction specificity is plotted, showing a positive trend. The regression line slopes upward, indicating increased specificity with distance. 6. Microtus pinetorum: Numerical interaction specificity is shown with a negative trend. The regression line slopes downward, indicating decreased specificity with distance. Each plot uses black dots to represent data points and a straight line to indicate the trend. No confidence bands are visible. The plots highlight how interaction specificity facets vary with distance to the equator across different species, with some showing positive relationships and others negative.

Navigate back to Figure 3.

## Figure 4 Long description

The image contains five scatter plots comparing interaction specificity with distance to the equator. Each plot features circular markers and a solid fitted line. The plots are labeled as follows: A) Afrotropical hosts with phylogenetic interaction specificity, B) Afrotropical fleas with functional interaction specificity, C) Palearctic hosts with functional interaction specificity, D) Palearctic fleas with numerical interaction specificity and E) Palearctic hosts with numerical interaction specificity. Plot A: The horizontal axis is Distance to equator, ranging from 3 to 9 and the vertical axis is Phylogenetic interaction specificity, ranging from minus 2.5 to 1.5. The fitted line slopes upward, indicating increasing specificity with distance. Plot B: The horizontal axis is Distance to equator, ranging from 3 to 9 and the vertical axis is Functional interaction specificity, ranging from minus 1.6 to 1.0. The fitted line slopes upward, showing increasing specificity with distance. Plot C: The horizontal axis is Distance to equator, ranging from 8.0 to 8.9 and the vertical axis is Functional interaction specificity, ranging from minus 2.0 to 2.0. The fitted line slopes downward, indicating decreasing specificity with distance. Plot D: The horizontal axis is Distance to equator, ranging from 8.0 to 8.9 and the vertical axis is Numerical interaction specificity, ranging from 0.0 to 0.6. The fitted line slopes upward, showing increasing specificity with distance. Plot E: The horizontal axis is Distance to equator, ranging from 8.0 to 9.0 and the vertical axis is Numerical interaction specificity, ranging from 0.0 to 0.5. The fitted line slopes upward, indicating increasing specificity with distance. Overall, Afrotropical hosts and fleas show increasing specificity with distance, while Palearctic hosts show decreasing functional specificity. Numerical specificity increases for both fleas and hosts in the Palearctic. The plots do not show multiple series and all markers are uniform in appearance. Notable clustering occurs at higher distances in Afrotropical panels.

Navigate back to Figure 4.

## Figure 5 Long description

The image A showing Afrotropical hosts. The x-axis is labeled Distance to equator, ranging from 4.0 to 8.5 in increments of 0.5. The y-axis is labeled Phylogenetic interaction specificity, ranging from minus 1.6 to 1.0 in increments of 0.2. A straight fitted line rises from left to right. Plotted points include approximately (4.0, minus 0.8), (4.5, minus 0.6), (5.0, minus 0.4), (6.0, minus 0.2), (7.0, 0.0), (7.5, 0.2), (8.0, 0.6) and (8.3, 0.9). The image B showing Afrotropical fleas. The x-axis is labeled Distance to equator, ranging from 4.0 to 8.5 in increments of 0.5. The y-axis is labeled Functional interaction specificity, ranging from minus 1.2 to 0.6 in increments of 0.2. A straight fitted line rises from left to right. Plotted points include approximately (4.0, minus 0.9), (4.5, minus 0.8), (5.5, minus 0.6), (6.5, minus 0.4), (7.5, minus 0.2), (8.0, 0.0) and (8.3, 0.4). The image C showing Palearctic fleas. The x-axis is labeled Distance to equator, ranging from 7.8 to 9.0 in increments of 0.2. The y-axis is labeled Functional interaction specificity, ranging from minus 1.4 to 1.0 in increments of 0.2. A straight fitted line falls from left to right. Plotted points include approximately (7.9, 0.4), (8.0, 0.2), (8.2, 0.0), (8.4, minus 0.4), (8.6, minus 0.6), (8.8, minus 0.8) and (9.0, minus 0.2). The image D showing Palearctic hosts. The x-axis is labeled Distance to equator, ranging from 7.8 to 9.0 in increments of 0.2. The y-axis is labeled Functional interaction specificity, ranging from minus 1.4 to 0.8 in increments of 0.2. A straight fitted line falls from left to right. Plotted points include approximately (7.9, 0.4), (8.1, 0.2), (8.3, 0.0), (8.5, minus 0.2), (8.7, minus 0.4), (8.9, minus 0.8) and (9.0, minus 1.0). The image E showing Afrotropical fleas. The x-axis is labeled Distance to equator, ranging from 4.0 to 8.5 in increments of 0.5. The y-axis is labeled Numerical interaction specificity, ranging from 0.10 to 0.30 in increments of 0.02. A straight fitted line rises from left to right. Plotted points include approximately (4.0, 0.12), (5.0, 0.14), (6.0, 0.16), (7.0, 0.20), (7.5, 0.22), (8.0, 0.24), (8.3, 0.28) and (8.5, 0.30). The image F showing Afrotropical hosts. The x-axis is labeled Distance to equator, ranging from 4.0 to 8.5 in increments of 0.5. The y-axis is labeled Numerical interaction specificity, ranging from 0.08 to 0.30 in increments of 0.02. A straight fitted line rises from left to right. Plotted points include approximately (4.0, 0.10), (5.0, 0.12), (6.0, 0.14), (7.0, 0.18), (7.5, 0.20), (8.0, 0.22), (8.3, 0.26) and (8.5, 0.30).

Navigate back to Figure 5.

## Table 3 Long description

The table lists host species with statistically significant relationships between distance from the equator (log-transformed) and three interaction-specificity facets: phylogenetic, functional, and numerical. For each species and facet, it reports the direction and strength of the relationship using a test statistic and the proportion of variance explained. Numerical specificity most often increases with greater distance from the equator, including multiple Afrotropical rodents (for example Gerbilliscus brantsii, Gerbilliscus leucogaster, and Rhabdomys pumilio), three Neotropical species, and Palearctic species such as Myodes rutilus and Sciurus vulgaris. Exceptions occur in the Nearctic, where Microtus pinetorum shows decreasing numerical specificity with distance, while Microtus pennsylvanicus and Tamias minimus increase. Phylogenetic specificity increases with distance for Afrotropical species (Gerbilliscus brantsii and Mus minutoides) and Nearctic Peromyscus leucopus, but decreases for Nearctic Tamias minimus and for Palearctic Craseomys rufocanus and Microtus oeconomus. Functional specificity increases for Afrotropical Gerbilliscus brantsii and Nearctic Microtus pennsylvanicus, but decreases for Palearctic Myodes glareolus and Myodes rutilus. Strength of fit varies widely, with some very strong relationships (for example Mus minutoides phylogenetic specificity) and others modest, so patterns should be interpreted as species-specific rather than universal across realms.

Navigate back to Table 3.

## Table 1 Long description

The table reports significant linear relationships between distance from the equator and three interaction-specificity facets (phylogenetic, functional, numerical) for fleas and hosts, summarized by t values, p values, and variance explained. In the Afrotropics, fleas show increasing functional and numerical specificity with distance, and hosts show increasing phylogenetic and numerical specificity; numerical relationships are relatively strong, explaining about 32 to 37 percent of variation. In the Nearctic, fleas show decreasing functional specificity but increasing numerical specificity, while hosts increase in phylogenetic, functional, and numerical specificity, each explaining about 19 to 25 percent of variation. In the Neotropics, only fleas show a significant increase in functional specificity, with a small fit (about 6 percent explained). In the Palearctic, fleas show decreasing functional specificity but increasing numerical specificity, and hosts show increasing numerical specificity, with host numerical specificity explaining about 28 percent of variation. Overall, numerical specificity most consistently increases with distance from the equator, while functional specificity can either increase or decrease depending on realm and whether fleas or hosts are analyzed. Only statistically significant relationships are included, so the absence of a row does not imply no effect, only that it was not significant in the underlying analysis.

Navigate back to Table 1.

## Table 2 Long description

The table lists flea species by biogeographic realm and reports significant relationships between distance from the equator (log transformed) and three interaction specificity facets: phylogenetic, functional, and numerical. For each species, it provides the direction and strength of the relationship plus model fit (R squared). In Afrotropics, Leptopsylla aethiopica shows phylogenetic specificity increasing with distance while functional specificity decreases; Chiastopsylla rossi shows a strong increase in numerical specificity with the highest fit (R squared 0.89). In the Nearctic, phylogenetic and functional specificity vary by species, with Orchopeas howardi and Orchopeas caedens decreasing with distance, while Ctenophthalmus pseudagyrtes increases slightly; numerical specificity decreases for Peromyscopsylla scotti but increases for Opisodasys keeni with a high fit (R squared 0.75). The Neotropics include one significant result: Polygenis atopus shows numerical specificity decreasing with distance with a moderately high fit (R squared 0.69). In the Palearctic, phylogenetic specificity increases for Amphipsylla sibirica, functional specificity shows mixed directions across species, and numerical specificity includes both increases (Amphipsylla sibirica, Leptopsylla taschenbergi) and decreases (Peromyscopsylla silvatica, Stenoponia tripectinata). Only species with statistically significant slopes are included, so the table does not represent all tested species or imply that nonlisted species lack relationships.

Navigate back to Table 2.

## Table 4 Long description

The table reports significant linear relationships between distance from the equator (log-transformed) and three interaction-specificity facets: phylogenetic, functional, and numerical, for fleas and hosts in two realms. In the Afrotropics, both fleas and hosts show increasing phylogenetic specificity with greater distance from the equator (t 3.44, p 0.001, R squared 0.23). Afrotropics also shows increasing numerical specificity with distance for both fleas and hosts (t 2.30, p 0.02, R squared 0.32). In the Palearctic, flea functional specificity decreases as distance increases (t minus 4.40, p less than 0.001, R squared 0.08). Palearctic numerical specificity increases with distance for fleas (t 2.86, p 0.01, R squared 0.21) and for hosts (t 2.33, p 0.02, R squared 0.28). Only statistically significant relationships are included, so the absence of a facet or realm does not imply no effect, only that it was not significant or not reported here.

Navigate back to Table 4.

## Table 5 Long description

The table reports linear model results linking distance from the equator (log-transformed) to two interaction specificity facets, functional and numerical, for fleas and hosts in two realms. In the Afrotropics, distance from the equator is positively associated with functional specificity for fleas (t 2.26, p 0.04, R squared 0.09) and hosts (t 2.41, p 0.03, R squared 0.22). Afrotropics numerical specificity also increases with distance for both fleas (t 2.96, p 0.02, R squared 0.39) and hosts (t 2.69, p 0.02, R squared 0.32). In the Palearctic, functional specificity decreases with distance for fleas (t minus 2.69, p 0.01, R squared 0.08) and hosts (t minus 2.76, p 0.01, R squared 0.10). Palearctic numerical specificity increases with distance for both fleas (t 3.21, p 0.001, R squared 0.21) and hosts (t 3.21, p less than 0.001, R squared 0.28). Overall, numerical specificity rises with distance in both realms, but functional specificity shows opposite directions between realms. Only statistically significant relationships are included, and several R squared values are modest, indicating limited explained variation in some models.

Navigate back to Table 5.
