# Supplementary material for: The latitudinal patterns of phylogenetic, functional and numerical interaction specificity in flea–mammal networks vary between biogeographic realms
Source: Parasitology. 2026 May 26;153(7):890–901. doi: 10.1017/S0031182026102182 (PMC13735568; doi:10.1017/S0031182026102182)
Supplement: Supplementary file 1 [file S0031182026102182sup001.docx]

**Appendix 1. Supplementary tables.**

**Table S1.** Numbers of regional networks, flea and host species interacting with at least three partners, and unique flea–host interactions in four biogeographic realms.

| Realm | Number of regional networks | Number of flea species | Number of host species | Number of unique interactions |
| --- | --- | --- | --- | --- |
| Afrotropics | 15 | 195 | 203 | 2437 |
| Nearctic | 23 | 203 | 251 | 3015 |
| Neotropics | 17 | 258 | 189 | 2414 |
| Palearctic | 36 | 212 | 329 | 4339 |

**Table S2**. Numbers of flea and host species interacting with at least three partners and occurring in at least six networks in the Afrotropics.

| Fleas | | Hosts | |
| --- | --- | --- | --- |
| Species | Number of networks | Species | Number of networks |
| *Chiastopsylla rossi* | 7 | *Aethomys chrysophilus* | 9 |
| *Ctenophthalmus calceatus* | 9 | *Aethomys namaquensis* | 8 |
| *Dinopsyllus ellobius* | 8 | *Cricetomys gambianus* | 8 |
| *Dinopsyllus lypusus* | 9 | *Crocidura hirta* | 6 |
| *Leptopsylla aethiopica* | 10 | *Cryptomys hottentotus* | 7 |
| *Listropsylla dorippae* | 6 | *Dendromus mystacalis* | 6 |
| *Xenopsylla nubica* | 7 | *Gerbilliscus brantsii* | 8 |
| *Xenopsylla versuta* | 7 | *Gerbilliscus leucogaster* | 9 |
|  |  | *Gerbillurus paeba* | 7 |
|  |  | *Grammomys dolichurus* | 7 |
|  |  | *Graphiurus murinus* | 6 |
|  |  | *Lophuromys flavopunctatus* | 7 |
|  |  | *Mastomys erythroleucus* | 6 |
|  |  | *Mastomys natalensis* | 14 |
|  |  | *Mus minutoides* | 7 |
|  |  | *Oenomys hypoxanthus* | 6 |
|  |  | *Otomys irroratus* | 6 |
|  |  | *Pelomys fallax* | 6 |
|  |  | *Rhabdomys pumilio* | 12 |
|  |  | *Saccostomus campestris* | 10 |
|  |  | *Thallomys paedulcus* | 7 |

**Table S3**. Numbers of flea and host species interacting with at least three partners and occurring in at least six networks in the Nearctic.

| Fleas | | Hosts | |
| --- | --- | --- | --- |
| Species | Number of networks | Species | Number of networks |
| *Aetheca wagneri* | 10 | *Blarina brevicauda* | 12 |
| *Amaradix bitterrootensis* | 6 | *Callospermophilus lateralis* | 6 |
| *Amaradix euphorbi* | 7 | *Cryptotis parva* | 7 |
| *Callistopsyllus terinus* | 7 | *Glaucomys sabrinus* | 10 |
| *Catallagia borealis* | 7 | *Glaucomys volans* | 14 |
| *Catallagia decipiens* | 7 | *Ictidomys tridecemlineatus* | 7 |
| *Ceratophyllus vison* | 14 | *Marmota flaviventris* | 7 |
| *Conorhinopsylla stanfordi* | 11 | *Marmota monax* | 11 |
| *Corrodopsylla curvata* | 13 | *Microtus montanus* | 6 |
| *Corrodopsylla hamiltoni* | 7 | *Microtus ochrogaster* | 6 |
| *Ctenophthalmus pseudagyrtes* | 16 | *Microtus pennsylvanicus* | 14 |
| *Doratopsylla blarinae* | 9 | *Microtus pinetorum* | 9 |
| *Epitedia cavernicola* | 6 | *Myodes gapperi* | 14 |
| *Epitedia wenmanni* | 21 | *Neotoma cinerea* | 6 |
| *Eumolpianus eumolpi* | 10 | *Neotoma floridana* | 6 |
| *Foxella ignota* | 10 | *Ochotona princeps* | 6 |
| *Hystrichopsylla dippiei* | 8 | *Onychomys leucogaster* | 8 |
| *Hystrichopsylla occidentalis* | 7 | *Peromyscus boylii* | 6 |
| *Malaraeus telchinus* | 8 | *Peromyscus leucopus* | 14 |
| *Megabothris abantis* | 8 | *Peromyscus maniculatus* | 21 |
| *Megabothris acerbus* | 6 | *Reithrodontomys megalotis* | 11 |
| *Megabothris asio* | 12 | *Scalopus aquaticus* | 8 |
| *Megabothris quirini* | 10 | *Sciurus carolinensis* | 13 |
| *Megarthroglossus divisus* | 6 | *Sciurus niger* | 10 |
| *Nearctopsylla genalis* | 8 | *Sigmodon hispidus* | 6 |
| *Opisodasys keeni* | 8 | *Sorex cinereus* | 8 |
| *Opisodasys pseudarctomys* | 11 | *Sorex palustris* | 6 |
| *Orchopeas caedens* | 12 | *Tamias minimus* | 9 |
| *Orchopeas howardi* | 15 | *Tamias striatus* | 11 |
| *Orchopeas leucopus* | 22 | *Tamiasciurus hudsonicus* | 17 |
| *Orchopeas sexdentatus* | 16 | *Thomomys talpoides* | 7 |
| *Oropsylla arctomys* | 11 | *Zapus hudsonius* | 8 |
| *Oropsylla idahoensis* | 8 |  |  |
| *Oropsylla tuberculata* | 7 |  |  |
| *Peromyscopsylla catatina* | 7 |  |  |
| *Peromyscopsylla hamifer* | 9 |  |  |
| *Peromyscopsylla hesperomys* | 17 |  |  |
| *Peromyscopsylla scotti* | 9 |  |  |
| *Peromyscopsylla selenis* | 8 |  |  |
| *Phalacropsylla allos* | 6 |  |  |
| *Rhadinopsylla fraterna* | 7 |  |  |
| *Rhadinopsylla sectilis* | 7 |  |  |
| *Stenoponia americana* | 15 |  |  |
| *Tamiophila grandis* | 7 |  |  |
| *Thrassis acamantis* | 6 |  |  |
| *Thrassis bacchi* | 8 |  |  |

**Table S4**. Numbers of flea and host species interacting with at least three partners and occurring in at least six networks in the Neotropics.

| Fleas | | Hosts | |
| --- | --- | --- | --- |
| Species | Number of networks | Species | Number of networks |
| *Adoratopsylla antiquorum* | 7 | *Cavia aperea* | 8 |
| *Adoratopsylla intermedia* | 10 | *Chironectes minimus* | 6 |
| *Craneopsylla minerva* | 14 | *Didelphis albiventris* | 7 |
| *Neotyphloceras crassispina* | 6 | *Didelphis aurita* | 7 |
| *Polygenis atopus* | 8 | *Eligmodontia typus* | 6 |
| *Polygenis axius* | 6 | *Galea musteloides* | 6 |
| *Polygenis bohlsi* | 11 | *Holochilus brasiliensis* | 7 |
| *Polygenis klagesi* | 7 | *Hylaeamys megacephalus* | 6 |
| *Polygenis occidentalis* | 6 | *Lutreolina crassicaudata* | 8 |
| *Polygenis platensis* | 7 | *Metachirus nudicaudatus* | 8 |
| *Polygenis pradoi* | 7 | *Nectomys squamipes* | 6 |
| *Polygenis rimatus* | 12 | *Oligoryzomys flavescens* | 8 |
| *Polygenis roberti* | 11 | *Oligoryzomys longicaudatus* | 7 |
| *Rhopalopsyllus lugubris* | 10 | *Oligoryzomys nigripes* | 7 |
|  |  | *Philander opossum* | 6 |
|  |  | *Phyllotis xanthopygus* | 8 |
|  |  | *Reithrodon auritus* | 7 |

**Table S5**. Numbers of flea and host species interacting with at least three partners and occurring in at least six networks in the Palearctic.

| Fleas | | Hosts | |
| --- | --- | --- | --- |
| Species | Number of networks | Species | Number of networks |
| *Amalaraeus penicilliger* | 22 | *Allactaga elater* | 7 |
| *Amphalius runatus* | 6 | *Apodemus agrarius* | 13 |
| *Amphipsylla rossica* | 17 | *Apodemus flavicollis* | 10 |
| *Amphipsylla schelkovnikovi* | 6 | *Apodemus peninsulae* | 6 |
| *Amphipsylla sibirica* | 11 | *Apodemus sylvaticus* | 12 |
| *Archaeopsylla erinacei* | 9 | *Apodemus uralensis* | 12 |
| *Atyphloceras nuperus* | 6 | *Arvicola amphibius* | 20 |
| *Callopsylla caspia* | 6 | *Chionomys nivalis* | 8 |
| *Callopsylla saxatilis* | 7 | *Cricetulus migratorius* | 12 |
| *Catallagia dacenkoi* | 8 | *Cricetus cricetus* | 9 |
| *Catallagia ioffi* | 6 | *Crocidura leucodon* | 6 |
| *Ceratophyllus indages* | 12 | *Crocidura russula* | 6 |
| *Ceratophyllus sciurorum* | 16 | *Crocidura suaveolens* | 9 |
| *Citellophilus tesquorum* | 10 | *Dryomys nitedula* | 8 |
| *Coptopsylla lamellifer* | 6 | *Glis glis* | 6 |
| *Corrodopsylla birulai* | 12 | *Meriones libycus* | 8 |
| *Ctenophthalmus agyrtes* | 9 | *Meriones meridianus* | 6 |
| *Ctenophthalmus assimilis* | 13 | *Micromys minutus* | 17 |
| *Ctenophthalmus bisoctodentatus* | 8 | *Microtus agrestis* | 15 |
| *Ctenophthalmus proximus* | 6 | *Microtus arvalis* | 23 |
| *Ctenophthalmus uncinatus* | 9 | *Microtus gregalis* | 6 |
| *Ctenophyllus armatus* | 7 | *Microtus oeconomus* | 14 |
| *Doratopsylla dasycnema* | 14 | *Microtus socialis* | 6 |
| *Frontopsylla elata* | 11 | *Microtus subterraneus* | 6 |
| *Hystrichopsylla talpae* | 18 | *Myodes glareolus* | 17 |
| *Leptopsylla taschenbergi* | 10 | *Myodes rufocanus* | 12 |
| *Megabothris calcarifer* | 8 | *Myodes rutilus* | 14 |
| *Megabothris rectangulatus* | 13 | *Myopus schisticolor* | 6 |
| *Megabothris turbidus* | 18 | *Neomys anomalus* | 6 |
| *Megabothris walkeri* | 10 | *Neomys fodiens* | 18 |
| *Mesopsylla tuschkan* | 7 | *Ochotona hyperborea* | 6 |
| *Myoxopsylla jordani* | 6 | *Sciurus vulgaris* | 15 |
| *Neopsylla acanthina* | 7 | *Sicista betulina* | 9 |
| *Neopsylla mana* | 9 | *Sorex araneus* | 18 |
| *Neopsylla pleskei* | 12 | *Sorex caecutiens* | 9 |
| *Neopsylla setosa* | 10 | *Sorex minutus* | 13 |
| *Nosopsyllus consimilis* | 8 | *Talpa europaea* | 14 |
| *Nosopsyllus laeviceps* | 7 | *Tamias sibiricus* | 10 |
| *Ophthalmopsylla volgensis* | 7 |  |  |
| *Oropsylla ilovaiskii* | 8 |  |  |
| *Oropsylla silantiewi* | 7 |  |  |
| *Palaeopsylla kohauti* | 8 |  |  |
| *Palaeopsylla soricis* | 19 |  |  |
| *Peromyscopsylla bidentata* | 15 |  |  |
| *Peromyscopsylla silvatica* | 10 |  |  |
| *Rhadinopsylla integella* | 15 |  |  |
| *Rhadinopsylla pentacantha* | 6 |  |  |
| *Stenoponia tripectinata* | 10 |  |  |
| *Tarsopsylla octodecimdentata* | 14 |  |  |
| *Xenopsylla conformis* | 9 |  |  |
